# Efficacy of Biologics for Surgical Treatment of Periodontal Suprabony Defects: A Systematic Review and Meta‐Analysis of Controlled Clinical Trials

**DOI:** 10.1111/jcpe.70078

**Published:** 2026-01-14

**Authors:** Sahar Baniameri, Parham Hazrati, Hamoun Sabri, Abdusalam E. Alrmali, Lucrezia Parma‐Benfenati, Saeed A. ElRefaei, Hom‐Lay Wang, Muhammad H. A. Saleh

**Affiliations:** ^1^ Department of Periodontics and Oral Medicine University of Michigan School of Dentistry Ann Arbor Michigan USA; ^2^ Center for Clinical Research and Evidence Synthesis in Oral Tissue Regeneration (CRITERION) Ann Arbor Michigan USA

**Keywords:** alveolar bone loss, dental enamel proteins, hyaluronic acid, periodontal pocket, periodontitis, platelet‐rich fibrin

## Abstract

**Aim:**

Limited bone support in suprabony defects hinders predictable regeneration, but adjunctive biologics can improve results. This systematic review and meta‐analysis aimed to evaluate the clinical efficacy of adjunctive biologics combined with open flap debridement (OFD) compared with OFD alone when treating suprabony defects.

**Materials and Methods:**

A systematic literature search was performed to identify controlled trials evaluating adult patients presenting horizontal periodontal defects. The primary outcome included residual probing depth (PD). Random‐effects meta‐analyses were performed; heterogeneity was assessed using *I* ^2^ statistics; risk of bias was evaluated using Cochrane RoB‐2; and evidence of quality was appraised via the GRADE framework.

**Results:**

Nine studies comprising 303 patients were included. Adjunctive use of biologics—including enamel matrix derivative (EMD), platelet‐rich fibrin (PRF) and hyaluronic acid (HA)—significantly enhanced clinical outcomes compared to OFD alone, with pooled mean differences favouring EMD and PRF for both residual PD (−0.89 and −0.42 mm) and clinical attachment level (CAL) (−1.32 and −0.69 mm). While subgroup differences were observed, notably with EMD, high heterogeneity was present for CAL and PD, and evidence of certainty ranged from low to very low.

**Conclusion:**

Adjunctive biologics improve clinical outcomes in suprabony periodontal defects; however, rigorous standardised trials are needed for reaching firm conclusions.

## 1 Introduction

The primary objective in treating periodontal defects is to halt disease progression and reduce or eliminate pockets, ideally by regenerating all lost periodontal tissues, including cementum, periodontal ligament and alveolar bone, rather than by repair through long junctional epithelium (Cortellini and Tonetti 2015); however, predictable regeneration remains challenging, especially in suprabony defects characterised by horizontal bone loss (Polimeni et al. 2006; Graziani et al. 2014). Suprabony periodontal defects differ structurally from intrabony defects by lacking supportive bone walls necessary for clot stabilisation, space maintenance and cellular migration (Polimeni et al. 2006; Wikesjö et al. 2008). Despite successfully reducing probing depths (PDs), traditional surgical techniques such as open flap debridement (OFD) often result in limited improvement in terms of clinical attachment level (CAL), gingival recession depth (REC) and patient discomfort (Graziani et al. 2014; Aimetti et al. 2017). These challenging defect configurations highlight a critical need for supplementary strategies to augment regenerative capacity. In recent decades, biologically active agents have emerged as a promising frontier in enhancing periodontal wound‐healing outcomes (Graziani et al. 2014).

Enamel matrix derivatives (EMDs), a purified protein complex derived from porcine developing enamel, have been extensively studied and shown to support regeneration by facilitating periodontal ligament fibroblast proliferation, cementogenesis and angiogenesis (Miron et al. 2016; Sculean et al. 2001). Similarly, platelet‐rich fibrin (PRF)—an autologous platelet concentrate rich in growth factors such as platelet‐derived growth factor (PDGF), transforming growth factor‐beta (TGF‐β) and vascular endothelial growth factor (VEGF)—has gained attention because of its capacity to enhance tissue regeneration and wound healing through sustained growth factor release, leukocyte‐mediated inflammation modulation and fibrin‐mediated clot stabilisation (Miron et al. 2017; Dohan Ehrenfest et al. 2009).

In line with growing clinical interest, several controlled clinical trials (CCTs) have evaluated the potential of adjunctive biologics in periodontal defect management, reporting varying degrees of clinical improvement in PD, CAL and REC (Di Tullio et al. 2013; Kizildağ et al. 2018; Jentsch and Purschwitz 2008; Bhutda and Deo 2013; Cortellini et al. 2022; Iorio‐Siciliano et al. 2021). Despite encouraging individual results, the existing literature presents considerable heterogeneity regarding surgical techniques, types of biologics and their application methods as well as outcome measures. Further, most available systematic reviews have primarily focused on intrabony or furcation defects (Tavelli et al. 2022), with comparatively limited evidence synthesised specifically for suprabony defects. Therefore, this systematic review and meta‐analysis aimed to assess the clinical efficacy of adjunctive biologics used in conjunction with surgical therapy for suprabony periodontal defects, with emphasis on residual PD, CAL and REC.

## 2 Materials and Methods

### 2.1 Protocol Registration and Reporting Format

This systematic review and meta‐analysis protocol was developed following the Preferred Reporting Items for Systematic Reviews and Meta‐Analyses (PRISMA) guidelines (Page et al. 2021). The review protocol was also registered in the International Prospective Register of Systematic Reviews (PROSPERO; United Kingdom; Registration ID: CRD420251035244).

### 2.2 PICOST Framework and Focus Question

The focus question guiding this study's primary objective was formulated based on the elements of the PICOST framework:

- *Population*: Adult patients (aged ≥ 18 years) diagnosed with periodontitis according to either the 1999 International Workshop for a Classification of Periodontal Diseases and Conditions (Armitage 1999) or the 2018 World Workshop by the EFP and AAP (Papapanou et al. 2018), presenting with at least one suprabony defect, who have completed phase I and II periodontal therapy.
- *Intervention*: Application of biological agents, including but not limited to EMD, autogenous blood‐derived products (e.g., PRF), recombinant human platelet–derived growth factor (rh‐PDGF) and hyaluronic acid (HA),in the surgical treatment of suprabony defects regardless of the flap design.
- *Comparison*: Conventional surgical approaches employing different surgical flap designs, such as papilla preservation techniques (PPTs), without the adjunctive use of biologics.
- *Outcomes*:
  ○ Primary outcome:
    - Residual PD
  ○ Secondary outcomes:
    - Residual CAL
    - Residual REC
    - Residual bleeding on probing (BOP) and plaque index (PI)
    - Radiographic (or clinical) bone‐level changes
    - Pocket closure.
- Study design: Controlled clinical trials (randomised or non‐randomised) employing either a parallel‐group or split‐mouth allocation design with at least five patients in each arm.
- Time: Trials with a minimum follow‐up period of 6 months.

The final focus question was formulated as follows: ‘What is the effect of employing biological agents such as EMD, PRF and HA in the surgical management of suprabony defects in patients diagnosed with periodontitis according to the 1999 or 2018 Workshop classification on primarily residual PD, and secondarily CAL, REC, BOP, PI and bone changes, in CCTs with at least 6 months of follow‐up’.

### 2.3 Eligibility Criteria

Inclusion criteria:

- Studies involving adult patients diagnosed with horizontal periodontal defects;
- Interventional studies evaluating regenerative techniques with EMD, platelet‐rich plasma (PRP), PRF, HA or other related biologics;
- Studies reporting quantitative clinical outcomes, such as residual PD, CAL, REC and radiographic bone changes.

Exclusion criteria:

- Animal studies, case reports, reviews and retrospective studies;
- Studies evaluating vertical defects and infrabony defects;
- Studies with a follow‐up period of less than 6 months.

### 2.4 Search Strategy

A comprehensive electronic search was conducted across PubMed/MEDLINE, Embase, Scopus and Web of Science. The search strategy employed both MeSH terms and free‐text keywords to ensure comprehensive coverage of relevant literature; no date, language or publication status restriction was applied. Hand‐searching was also performed on the *Journal of Periodontology*, the *Journal of Periodontal Research*, and the *Journal of Clinical Periodontology* up to June 2025, and the bibliographies of all retrieved papers and review articles. The finalised search strategy for each database is provided in Table S1.

### 2.5 Study Selection and Data Extraction

Two independent reviewers (S.B. and H.S.) screened titles and abstracts for relevance. Full‐text articles meeting the inclusion criteria were assessed independently by both reviewers. Disagreements were resolved by consultation with a third reviewer (M.H.A.S.). Subsequently, data were extracted using a structured data collection form, which included the following:

- Study characteristics (e.g., author, publication year, study design, sample size)
- Intervention details (e.g., the material used, surgical technique, follow‐up period)
- Outcome data (e.g., PD, CAL, radiographic bone change, complications)

### 2.6 Risk of Bias Assessment

The risk of bias in the included randomised controlled trials (RCTs) and CCT was assessed using the Cochrane Collaboration's Risk of Bias (RoB2) and Risk of Bias in Non‐randomised Studies of Interventions (ROBINS‐I) tools, respectively. Each study was scored across the domains specified in each tool and received an overall score of ‘low’, ‘moderate’ or ‘high’ risk of bias based on these assessments (Sterne et al. 2016, 2019).

### 2.7 Certainty of Evidence

The strength of evidence for each meta‐analytic outcome was evaluated using the Grading of Recommendations, Assessment, Development, and Evaluation (GRADE) framework (Balshem et al. 2011). In this framework, the risk of bias of the included studies, inconsistencies (heterogeneity) among the studies, indirectness and imprecision were considered. Significant issues in any of these domains reduced the certainty of evidence by one level.

### 2.8 Meta‐Analysis

A single author (P.H.) conducted pairwise meta‐analyses in RStudio (version 2024.12.1 + 563, PBC) (meta, metafor) using a generic inverse‐variance random‐effects model to compare OFD plus biologics with OFD alone for residual PD, CAL and REC (Balduzzi et al. 2019; Takeshima et al. 2014). Between‐study heterogeneity was quantified using *I* ^2^ (from Cochran's *Q*) and *τ* ^2^, with statistical significance set at 0.05. Robustness was evaluated using leave‐one‐out sensitivity analysis (for additional information, see the Appendix).

## 3 Results

### 3.1 Study Selection

**FIGURE 1:**
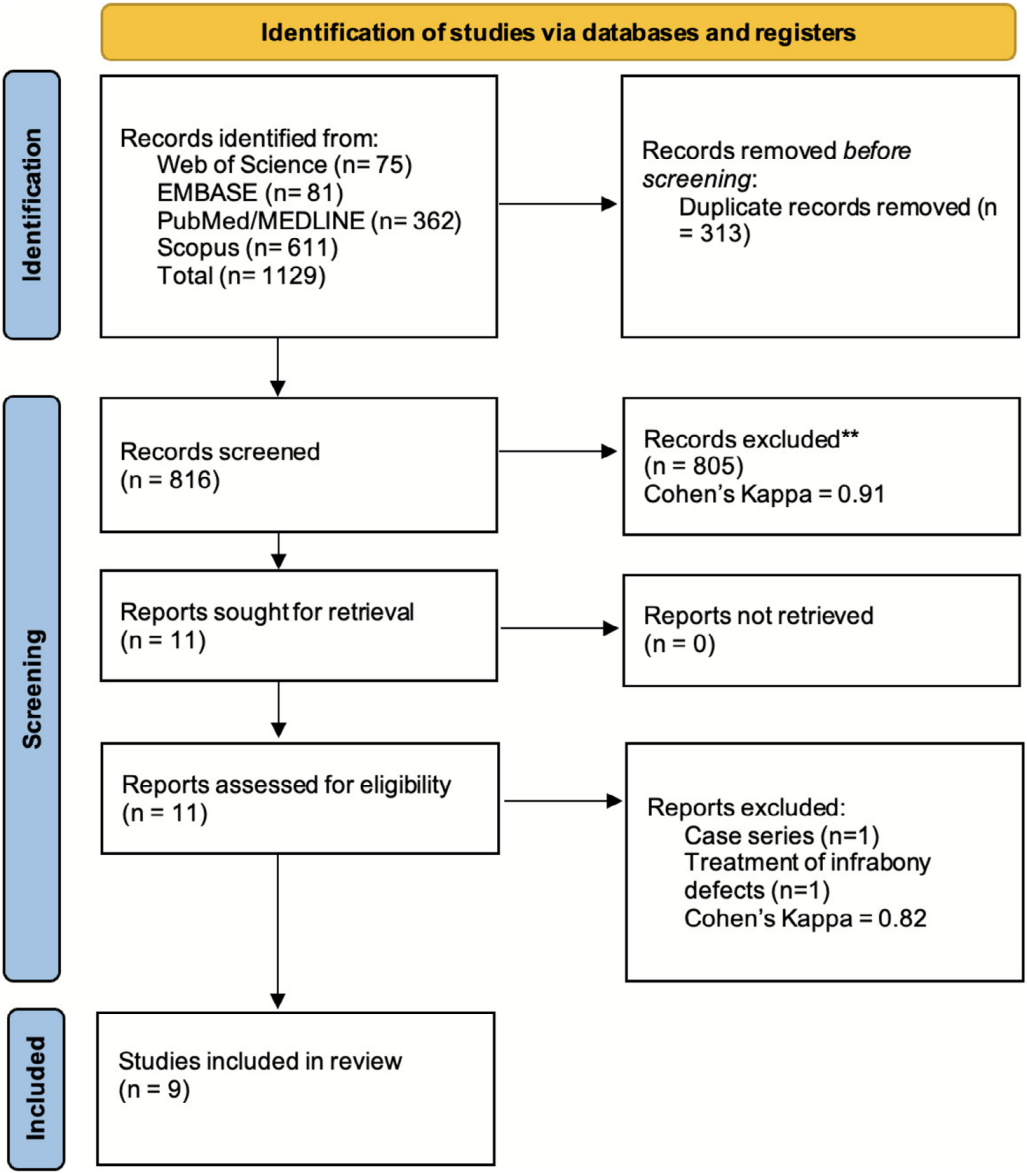
PRISMA flowchart of the review.

After combining citations from all databases and removing duplicates, a total of 816 records were selected according to their titles and abstracts (Figure 1). Eleven studies were identified for full‐text review, all of which were successfully accessed and retrieved. Among these, nine studies met the eligibility criteria and were included in the final review, while two were excluded (Table S2). Inter‐reviewer reliability was high, with a percent agreement of 95.70% and Cohen's Kappa of 0.91 (95% CI: 0.88–0.95) during the title and abstract screening phase, and 90.91% and 0.82 (95% CI: 0.48–1.00) at the full‐text review stage.

### 3.2 Study Characteristics

All the included studies were published between 2003 and 2025. Five studies followed a parallel‐arm design (Di Tullio et al. 2013; Jentsch and Purschwitz 2008; Iorio‐Siciliano et al. 2021; Vela et al. 2024; Subramanian et al. 2025), while four employed a split‐mouth design (Kizildağ et al. 2018; Joseph et al. 2014; Yilmaz et al. 2003; Debnath and Chatterjee 2018). The total number of patients included across the studies was 303, with sample sizes ranging from 8 to 65 participants. The follow‐up duration varied, ranging from 6 (Kizildağ et al. 2018; Subramanian et al. 2025) to 12 months (Di Tullio et al. 2013; Jentsch and Purschwitz 2008; Iorio‐Siciliano et al. 2021; Vela et al. 2024), while three studies assessed outcomes at 8 or 9 months (Joseph et al. 2014; Yilmaz et al. 2003; Debnath and Chatterjee 2018).

Four of the included studies evaluated EMD (Di Tullio et al. 2013; Jentsch and Purschwitz 2008; Iorio‐Siciliano et al. 2021; Yilmaz et al. 2003), and four studied PRF (Subramanian et al. 2025; Joseph et al. 2014; Debnath and Chatterjee 2018; Kizildağ et al. 2018). Only one study assessed the application of HA (Vela et al. 2024).

OFD was the most used baseline procedure, combined with adjunctive therapies such as EMD, PRF gel, PRF membranes or HA. Control groups in all studies involved OFD alone or variations of surgical incisions without adjunctive therapies. A summary of the characteristics of the included studies is presented in Tables 1 and 2.

**TABLE 1:** Overview of the outcomes reported in the included studies.

Author (year)	Groups (*N*)	Baseline PD (mm)	PD (mm)	PD reduction (mm)	Baseline CAL (mm)	CAL (mm)	CAL gain (mm)	Baseline REC (mm)	REC (mm)	REC increase (mm)
Yilmaz et al. (2003)	Intracrevicular incision + EMP (PD > 4 mm) (10)	5.08 ± 0.63	2.2 ± 0.48	2.87 ± 0.76	NA	NA	2.16 ± 0.60	3.68 ± 1.57	4.36 ± 1.29	0.68 ± 0.48
	Intracrevicular incision (PD > 4 mm) (10)	5.11 ± 0.48	3.57 ± 0.89	1.53 ± 0.82	NA	NA	0.54 ± 0.79	3.68 ± 1.19	4.78 ± 1.15	1.06 ± 0.49
	Reverse bevel incision + EMP (PD > 4 mm) (10)	5.27 ± 0.45	2.36 ± 0.37	2.92 ± 0.44	NA	NA	2.27 ± 0.63	3.64 ± 0.40	4.31 ± 0.72	0.67 ± 0.59
	Reverse bevel incision (PD > 4 mm) (10)	5.30 ± 0.74	2.38 ± 0.47	2.87 ± 0.97	NA	NA	1.56 ± 1.50	3.60 ± 0.93	4.64 ± 1.32	1.35 ± 0.75
Jentsch and Purschwitz (2008)	OFD + EMD (25)	4.30 ± 0.95	2.75 ± 0.44	1.55 ± 0.90	4.92 ± 1.18	3.95 ± 0.97	0.97 ± 0.92	NA	NA	NA
	OFD (14)	4.07 ± 0.67	3.66 ± 0.83	0.41 ± 0.66	4.41 ± 0.82	4.34 ± 1.03	0.07 ± 0.55	NA	NA	NA
Di Tullio et al. (2013)	SPPF + EMD (25)	5.96 ± 0.84	2.48 ± 0.65	3.48 ± 0.77	6.76 ± 1.01	3.96 ± 0.67	2.80 ± 0.86	0.83 ± 0.70	1.48 ± 0.82	0.68 ± 0.47
	SPPF (25)	6.4 ± 1.0	4.12 ± 0.83	2.28 ± 0.89	7.16 ± 1.10	6.12 ± 0.88	1.04 ± 0.61	0.79 ± 0.58	2.0 ± 0.64	1.24 ± 0.72
Joseph et al. (2014)	OFD + PRF gel + PRF membrane (15)	4.43 ± 0.59	2.73 ± 0.45	1.70 ± 0.45	4.40 ± 1.25	2.7 ± 1.14	1.70 ± 0.52	0.60 ± 0.47	0.63 ± 0.48	0.03 ± 0.12
	OFD + PRF gel (15)	4.33 ± 0.59	2.60 ± 0.63	1.73 ± 0.53	4.83 ± 1.11	3.26 ± 1.19	1.56 ± 0.62	0.96 ± 0.51	1.03 ± 0.69	0.06 ± 0.25
	OFD (15)	4.3 ± 0.41	3.20 ± 0.41	1.10 ± 0.38	4.23 ± 1.47	3.37 ± 1.32	0.86 ± 0.58	0.73 ± 0.75	0.83 ± 0.74	0.10 ± 0.20
Kizildağ et al. (2018)	OFD + L‐PRF (16)	6.12 ± 1.12	2.73 ± 1.26	3.39 ± 1.19	5.50 ± 0.92	2.60 ± 0.70	2.90 ± 0.81	NA	NA	NA
	OFD (16)	5.87 ± 0.60	3.55 ± 1.07	2.32 ± 0.86	5.54 ± 1.11	3.55 ± 0.85	1.99 ± 0.98	NA	NA	NA
Debnath and Chatterjee (2018)	OFD + IMP + PRFM (8)	6.79 ± 1.10	5.21 ± 0.96	NA	5.79 ± 1.10	4.29 ± 0.85	NA	NA	NA	NA
	OFD (8)	6.79 ± 1	5.75 ± 0.89	NA	5.89 ± 0.99	5.04 ± 0.84	NA	NA	NA	NA
Iorio‐Siciliano et al. (2021)	OFD + EMD (36)	6.4 ± 0.5	2.4 ± 0.5	3.90 ± 0.60	6.7 ± 0.5	3.3 ± 0.9	3.40 ± 0.60	0.2 ± 0.4	1.0 ± 1.1	0.5 ± 0.7
	OFD (29)	6.3 ± 0.6	3.0 ± 0.5	3.20 ± 0.60	6.7 ± 0.7	4.9 ± 0.6	1.80 ± 0.60	0.4 ± 0.8	1.8 ± 0.7	1.40 ± 1.00
Vela et al. (2024)	OFD + HA (30)	5.83 ± 1.31	2.55 ± 0.94	3.28 ± 1.14	6.31 ± 1.44	3.25 ± 1.5	3.06 ± 1.13	1.16 ± 1.4	1.08 ± 1.36	0.08 ± 0.76
	OFD (30)	5.9 ± 1.28	3.29 ± 1.05	2.61 ± 1.22	5.92 ± 1.66	4.48 ± 1.42	1.44 ± 1.07	0.95 ± 1.1	1.69 ± 1.25	0.74 ± 1.03
Subramanian et al. (2025)	SPPF + PRF (15)	6.53 ± 1.24	3.93 ± 0.25	NA	8.73 ± 1.33±	6.6 ± 0.82	NA	2.20 ± 0.77	2.67 ± 0.72	NA
	SPPF (15)	6.8 ± 1.42	4.07 ± 0.59	NA	8.47 ± 1.4±	6.93 ± 1.48	NA	1.67 ± 0.61	2.87 ± 0.99	NA

*Note*: All the presented values are represented as the mean ± standard deviation (SD). *p < 0.05, **p < 0.01, ***p < 0.001.

**TABLE 2:** Summary of the included controlled clinical trials.

Author (year)	Design	Country	Funding	Groups (*N*)	Male/female	Age	Smoker	Follow‐up	Incisions	Biologics	Bleeding indices	Plaque indices
Yilmaz et al. (2003)	RCT (split mouth)	Turkey	BIORA AB, Sweden	Intracrevicular incision + EMP (PD > 4 mm)	6/14	45.5	0	8 months	Intracrevicular incision	Emdog ain (BIORA AB)	0.14 ± 0.14	0.36 ± 0.43
				Intracrevicular incision (PD > 4 mm)							0.42 ± 0.42	0.42 ± 0.33
				Reverse bevel incision + EMP (PD > 4 mm)					Reverse bevel incision		0.09 ± 0.16	0.33 ± 0.23
				Reverse bevel incision (PD > 4 mm)							0.21 ± 0.39	0.47 ± 0.35
Jentsch and Purschwitz (2008)	RCT (parallel)	Germany	None	OFD + EMD (25)	5/20	44.4 ± 8.8	NR	12 months	Intra‐sulcular incision	Emdogain (Straumann)	0.19 ± 0.16	NA
				OFD (14)	5/9	52.1 ± 9.2				NA	0.29 ± 0.19	
Di Tullio et al. (2013)	RCT (parallel)	Italy	University funding	SPPF + EMD (25)	10/15	53.3 ± 5.7	0	12 months	Intracrevicular Incision	Emdogain (Straumann)	NA	NA
				SPPF (25)	9/14	51.2 ± 5.2				NA		
Joseph et al. (2014)	Non‐randomised clinical trial (split mouth)	India	None	OFD + PRF gel + PRF membrane (15)	6/9	36.3 ± 6.44	0	9 months	Crevicular incision	PRF gel + PRF membrane	NA	NA
				OFD + PRF gel (15)						PRF gel		
				OFD (15)						NA		
Kizildağ et al. (2018)	RCT (split mouth)	Turkey	University funding	OFD + L‐PRF (16)	9/7	37.51 ± 7.48	0	6 months	Sulcular incision	L‐PRF	0.23 ± 0.02	0.22 ± 0.02
				OFD (16)						NA	0.23 ± 0.02	0.23 ± 0.02
Debnath and Chatterjee (2018)	RCT (split mouth)	India	None	OFD + IMP + PRFM OFD	NA	NA	NA	9 months	Crevicular incision	PRFM gel	NA	0.62 ± 0.23
											NA	0.66 ± 0.26
Iorio‐Siciliano et al. (2021)	RCT (parallel)	Italy	None	OFD + EMD (36)	15/21	49.7 ± 8.6	5	12 months	MMPT/SPPT	Emdogain (Straumann)	20.4 ± 3.4	21.9 ± 3
				OFD (29)	14/15	49.3 ± 7.5	5			NA	19.9 ± 2.90	21.1 ± 2.4
Vela et al. (2024)	RCT (parallel)	Romania	University funding	OFD + HA	18/12	49.8 ± 7.4	8	12 months	NA	Cross‐linked HA gel (Bioscience)	16.40 ± 2.10	19.00 ± 2.00
				OFD	16/14	46.9 ± 8.1	6			NA	17.70 ± 2.40	19.30 ± 2.00
Subramanian et al. (2025)	RCT (parallel)	India	None	SPPF + PRF	9/6	38.4 ± 8.9	0	6 months	Intracrevicular incision	PRF matrix	16.00 ± 1.06	16.27 ± 1.83
				SPPF	6/9	37.8 ± 7.06				NA	16.93 ± 1.62	16.67 ± 1.83

### 3.3 Results of Individual Studies

#### 3.3.1 Baseline and Treatment Characteristics of the Suprabony Defects

The baseline characteristics across the included studies were generally comparable. No statistically significant differences were reported in demographic variables such as age, gender and smoking habits between the test and control groups. Studies by Iorio‐Siciliano et al. (2021), Kizildağ et al. (2018), and Jentsch and Purschwitz (2008) confirmed that PD, CAL, REC and bone levels were similar between groups at baseline. Yilmaz et al. (2003) and Subramanian et al. (2025) used relative attachment level (RAL) instead of CAL, calculated using a stent as a reference point. All included patients underwent steps 1 and 2 of periodontal treatment, including supragingival biofilm control and oral hygiene instructions in step 1 and subgingival instrumentation in step 2, prior to surgical intervention (for more information, see the Appendix).

#### 3.3.2 Probing Depth

##### 3.3.2.1 Enamel Matrix Derivative

Three out of four studies assessing EMD (Di Tullio et al. 2013; Jentsch and Purschwitz 2008; Iorio‐Siciliano et al. 2021) reported significantly lower residual PD, as well as higher PD reduction, for test groups compared to OFD controls (*p* < 0.05). In the study by Yilmaz et al. (2003), residual PD values were presented descriptively, and statistical testing was limited to PD reduction, which showed a significant advantage over the control treatment with reverse bevel incision. In the EMD‐treated arms, average residual PD consistently ranged from 2.20 to 2.75 mm. PD reduction also ranged from 1.55 (Di Tullio et al. 2013) to 3.9 mm (Jentsch and Purschwitz 2008), on average. Both studies implementing papilla preservation or conservative flap designs (Di Tullio et al. 2013; Iorio‐Siciliano et al. 2021) demonstrated significant differences favouring the EMD groups.

##### 3.3.2.2 Platelet‐Rich Fibrin

Except for the study by Subramanian et al. (2025), where no significant difference in residual PD was observed between groups, the other three studies evaluating PRF (Joseph et al. 2014; Debnath and Chatterjee 2018; Kizildağ et al. 2018) reported either significantly lower residual PD (Debnath and Chatterjee 2018; Kizildağ et al. 2018) or significantly higher PD reduction (Joseph et al. 2014) in the PRF groups compared to their respective controls (*p* < 0.05). Residual PD after treatment with PRF ranged from 2.66 to 5.21 mm. Joseph et al. (2014) observed a PD reduction of 1.73 mm in the PRF gel group and 1.7 mm in the PRF gel plus PRF membrane group, while the control group exhibited a smaller reduction of 1.1 mm (*p* < 0.001).

##### 3.3.2.3 Hyaluronic Acid

At 12 months, Vela et al. (2024) found significantly lower residual PD with HA plus OFD than with OFD alone (2.55 ± 0.94 vs. 3.29 ± 1.05; *p* = 0.032). Also, both groups showed significant improvement from baseline (*p* < 0.0001).

#### 3.3.3 Clinical Attachment Level and Relative Attachment Level

##### 3.3.3.1 Enamel Matrix Derivative

Among the four studies evaluating EMD (Di Tullio et al. 2013; Iorio‐Siciliano et al. 2021; Jentsch and Purschwitz 2008; Yilmaz et al. 2003), lower residual CAL as well as greater CAL gain in the test groups compared to OFD alone was reported in two studies (*p* < 0.05) (Di Tullio et al. 2013; Iorio‐Siciliano et al. 2021). Likewise, Yilmaz et al. (2003) only detected a significantly higher RAL gain but did not test residual RAL statistically. While Jentsch and Purschwitz (2008) observed no difference in residual CAL between groups, they detected a significantly greater CAL gain with EMD (Jentsch and Purschwitz 2008). While CAL/RAL gain values ranged from 0.97 to 3.4 mm, the average residual CAL was consistently between 3.3 and 3.96 mm among the studies. Similar to PD results, both studies employing minimally invasive or papilla‐preservation techniques reported significant improvements favouring EMD.

##### 3.3.3.2 Platelet‐Rich Fibrin

Consistent with the findings on PD, significantly higher CAL gain (Joseph et al. 2014) or lower residual CAL (Debnath and Chatterjee 2018; Kizildağ et al. 2018) was reported in three studies (Joseph et al. 2014; Kizildağ et al. 2018; Subramanian et al. 2025), while Subramanian et al. (2025) did not find any significant difference between PRF and OFD in terms of residual CAL. Residual CAL in PRF‐treated sites varied from 2.6 to 6.6 mm, on average. Also, Joseph et al. (2014) reported nearly identical CAL gain to PD reduction, suggesting minimal REC increase.

##### 3.3.3.3 Hyaluronic Acid

Both groups showed statistically significant CAL again at 12 months (control: 1.44 ± 1.07, test: 3.06 ± 1.13). The intergroup comparison also showed significantly lower residual CAL in the test group compared to the control group (2.55 ± 0.94 vs. 3.29 ± 1.05; *p* < 0.0001).

#### 3.3.4 Recession Depth

See the Appendix.

#### 3.3.5 Bone‐Level Changes

See the Appendix.

#### 3.3.6 Bleeding and Plaque Indices

See the Appendix.

#### 3.3.7 Meta‐Analysis

##### 3.3.7.1 Probing Depth

**FIGURE 2:**
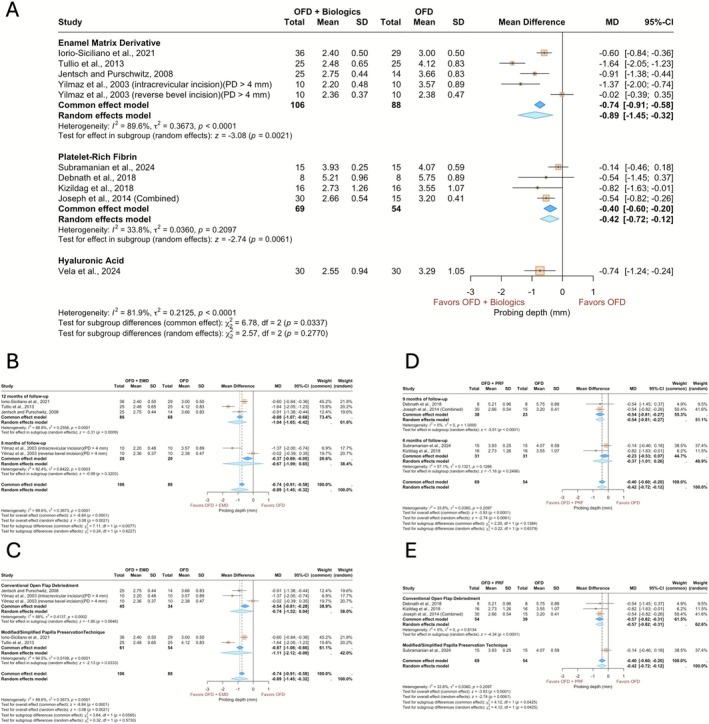
Forest plots summarising the outcome of the change in probing pocket depths. (A) Sub‐analysis based on the biologics applied. (B) Sub‐analysis based on follow‐up time for EMD. (C) Sub‐analysis based on the surgical technique for EMD. (D) Sub‐analysis based on follow‐up time for PRF. (E) Sub‐analysis based on surgical techniques for PRF. The models depict both common and random effects.

Nine studies comprising 10 treatment arms and their corresponding controls, with a total of 303 patients, were included in the meta‐analysis for residual PD (Figure 2A). The pooled estimates showed a significantly lower residual PD for both EMD and PRF compared to OFD alone, with a mean difference (MD) of −0.89 (95% CI: −1.45 to −0.32, *p* = 0.0021) for EMD and −0.42 (95% CI: −0.72 to −0.12, *p* = 0.0061) for PRF. Notably, the studies using EMD exhibited considerable heterogeneity (*I* ^2^ = 89.6%, *τ* ^2^ = 0.37, *p* < 0.0001). However, no statistically significant difference was found between the treatment effects of the two biologics (*p* = 0.2770). When grouped by the surgical technique (Figure 2C), EMD showed favourably lower residual PD across the PPT approach, with significantly lower residual PD noted compared to controls, but no difference was detected between conventional OFD with or without EMD. The effect size was −1.11 (95% CI: −2.12 to −0.09, *p* = 0.0333) for the PPT technique and −0.74 (95% CI: −1.52 to 0.04, *p* = 0.0646) for conventional OFD, although the between‐group difference was not significant (*p* = 0.5730). Surgical technique–based subgroup analysis indicated that the use of PRF with conventional OFD led to a significantly lower PD (−0.57, 95% CI: −0.82 to −0.31, *p* < 0.0001) (Figure 2E), and this effect was significantly more pronounced than in the PPT subgroup (*p* = 0.0425) (for more information, see the Appendix).

##### 3.3.7.2 Clinical Attachment Level

**FIGURE 3:**
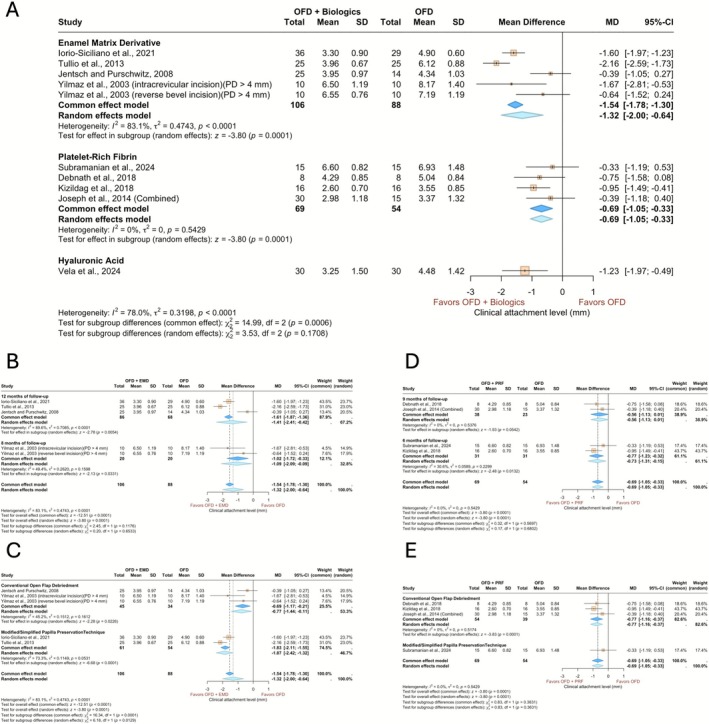
Forest plots summarising the outcome of the clinical attachment level gain. (A) Sub‐analysis based on the biologics applied. (B) Sub‐analysis based on the follow‐up time for EMD. (C) Sub‐analysis based on the surgical technique for EMD. (D) Sub‐analysis based on the follow‐up time for PRF. (E) Sub‐analysis based on the surgical techniques for PRF. The models depict both common and random effects.

The same pool of studies, trial arms and patients used for the meta‐analysis of PD was used for the meta‐analysis of residual CAL (Figure 3). The meta‐analysis revealed an MD of −1.32 (95% CI: −2.00 to −0.64) and −0.69 (95% CI: −1.05 to −0.33) in favour of OFD with EMD and PRF over OFD alone, respectively (Figure 3A), both of which represented a significantly lower CAL with biologics (*p* = 0.0001). Heterogeneity was significant across the studies employing EMD (*I* ^2^ = 83.1%, *τ* ^2^ = 0.47, *p* < 0.0001), indicating considerable variability in effect estimates. Treatment effect did not vary significantly between different biologics (*p* = 0.1708). Also, another subgroup analysis of studies using EMD stratified by surgical technique (Figure 3C) showed consistently lower residual CAL with the use of adjunct biologics, regardless of the technique. Both the OFD (−0.77, 95% CI: −1.44 to −0.11, *p* = 0.0226) and PPT (−1.87, 95% CI: −2.42 to −1.32, *p* < 0.0001) subgroups showed significantly lower residual CAL values compared to controls, with a notably higher effect observed in the PPT subgroup (*p* = 0.0129).

Similarly, when stratified by the surgical technique (Figure 3E), PRF showed significantly lower CAL values with OFD (−0.77, 95% CI: −1.16 to 0.37, *p* = 0.0001), with no significant difference between the OFD and PPT subgroups (*p* = 0.3631) (for more information, see the Appendix).

##### 3.3.7.3 Recession Depth

**FIGURE 4:**
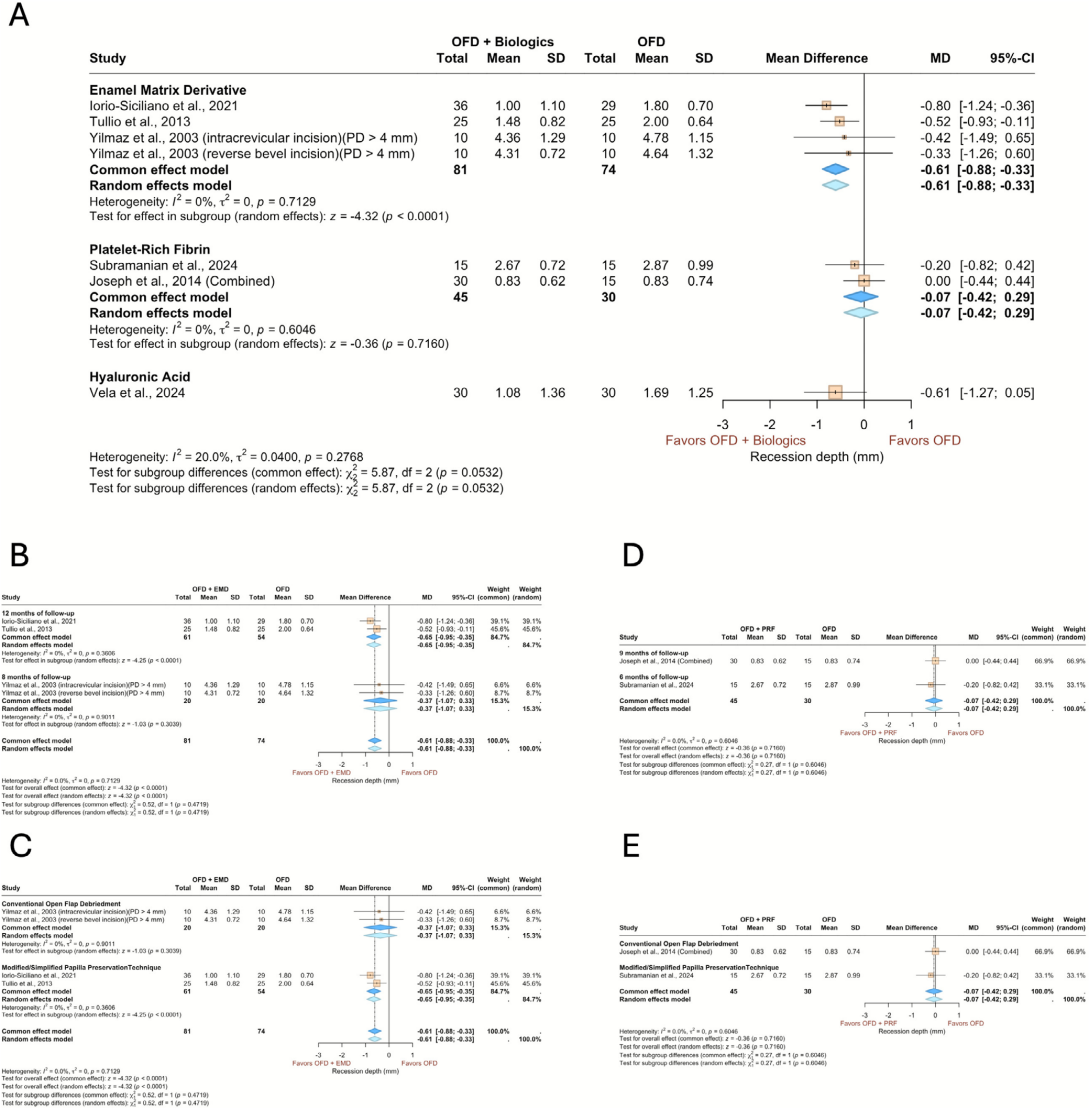
Forest plots summarising the outcome of the increase in the recession depth. (A) Sub‐analysis based on the biologics applied. (B) Sub‐analysis based on the follow‐up time for EMD. (C) Sub‐analysis based on the surgical technique for EMD. (D) Sub‐analysis based on the follow‐up time for PRF. (E) Sub‐analysis based on the surgical techniques for PRF. The models depict both common and random effects.

Six studies comprising seven treatment arms and their corresponding controls, totalling 215 patients, were included in the meta‐analysis for residual REC (Figure 4A). The pooled estimates indicated a significantly lower residual REC for EMD compared to OFD alone (−0.61, 95% CI: −0.88 to −0.33, *p* < 0.0001), while no significant difference was observed for PRF (−0.07, 95% CI: −0.42 to 0.29, *p* = 0.7160). The comparison between the two biologics did not yield a statistically significant difference (*p* = 0.0532). Subgroup analysis by surgical technique (Figure 4C) showed a significantly lower REC when EMD was used in conjunction with PPT (−0.65, 95% CI: −0.95 to −0.35, *p* < 0.001), while no significant benefit was seen with conventional OFD (−0.37, 95% CI: −1.07 to 0.33, *p* = 0.3039). The between‐technique comparison did not reveal a statistically meaningful difference (*p* = 0.4719). No significant differences were detected between follow‐up durations or surgical techniques in PRF studies (*p* = 0.6046) (for more information, see the Appendix).

##### 3.3.7.4 Plaque and Bleeding Indices

See the Appendix and Figure S1.

##### 3.3.7.5 Sensitivity Analysis

As presented in Table 3, the results of residual CAL, PD and REC meta‐analyses remained consistent across all iterations in EMD, indicating that the findings were generally stable. However, the overall effect estimates of PRF on residual CAL and PD changed and became statistically insignificant when Kizildağ et al. (2018) and Joseph et al. (2014) were excluded from the analysis, respectively. Changes in the subgroup analysis results following the leave‐one‐out analysis are presented in the Appendix.

**TABLE 3:** Sensitivity analysis with the leave‐one‐out approach, assessing the sensitivity of the overall effect size, subgroup differences according to time and subgroup differences according to the type of biological used. Bold values indicate a shift in significance.

Outcome	Biologic	Omitted study	Intra‐subgroup			Inter‐subgroup		
			Biologic subgroup	Surgical technique subgroup	Time subgroup	Biologic subgroup	Surgical technique subgroup	Time subgroup
CAL	EMD	Iorio‐Siciliano et al. (2021)	Significant	Significant	**Insignificant**	Insignificant	Significant	Insignificant
		Di Tullio et al. (2013)	Significant	Significant	**Insignificant**	Insignificant	Significant	Insignificant
		Jentsch and Purschwitz (2008)	Significant	Significant	Significant	Insignificant	**Insignificant**	Insignificant
		Yilmaz et al. (2003)	Significant	**Insignificant**	NA	Insignificant	Significant	NA
	PRF	Subramanian et al. (2025)	Significant	NA	Significant	Insignificant	NA	Insignificant
		Debnath and Chatterjee (2018)	Significant	Significant	Insignificant	Insignificant	Insignificant	Insignificant
		Kizildağ et al. (2018)	**Insignificant**	**Insignificant**	**Insignificant**	Insignificant	Insignificant	Insignificant
		Joseph et al. (2014)	Significant	Significant	NA	Insignificant	Insignificant	NA
	HA	Vela et al. (2024)	NA	NA	NA	Insignificant	NA	NA
PD	EMD	Iorio‐Siciliano et al. (2021)	Significant	Significant	Significant	Insignificant	**Significant**	Insignificant
		Di Tullio et al. (2013)	Significant	Significant	Significant	Insignificant	Insignificant	Insignificant
		Jentsch and Purschwitz (2008)	Significant	Insignificant	Significant	Insignificant	Insignificant	Insignificant
		Yilmaz et al. (2003)	Significant	**Significant**	NA	Insignificant	Insignificant	NA
	PRF	Subramanian et al. (2025)	Significant	NA	**Significant**	Insignificant	NA	Insignificant
		Debnath and Chatterjee (2018)	Significant	Significant	Significant	Insignificant	Significant	Insignificant
		Kizildağ et al. (2018)	Significant	Significant	Insignificant	Insignificant	**Insignificant**	Insignificant
		Joseph et al. (2014)	**Insignificant**	Significant	Insignificant	Insignificant	**Insignificant**	Insignificant
	HA	Vela et al. (2024)	NA	NA	NA	Insignificant	NA	NA
REC	EMD	Iorio‐Siciliano et al. (2021)	Significant	Significant	Significant	Insignificant	Insignificant	Insignificant
		Di Tullio et al. (2013)	Significant	Significant	Significant	Insignificant	Insignificant	Insignificant
		Yilmaz et al. (2003)	Significant	NA	NA	**Significant**	NA	NA
	PRF	Subramanian et al. (2025)	Insignificant	NA	NA	Insignificant	NA	NA
		Joseph et al. (2014)	Insignificant	NA	NA	Insignificant	NA	NA
	HA	Vela et al. (2024)	NA	NA	NA	**Significant**	NA	NA

##### 3.3.7.6 Quality of Evidence

See the Appendix and Table S3.

### 3.4 Risk of Bias Assessment

The only CCT included in this review had a low overall risk of bias (Figure S2). Also, as shown in Figure S3, the risk of bias assessment indicated that only two of the included RCTs had an overall risk of bias categorised as ‘low’ (Di Tullio et al. 2013; Iorio‐Siciliano et al. 2021). Debnath and Chatterjee (2018)'s was the only RCT rated as having ‘some concerns’ due to issues with the randomisation process. Additionally, four RCTs showed some concerns related to deviations from the intended intervention, and one RCT raised concerns about missing outcome data (Vela et al. 2024).

## 4 Discussion

This systematic review and meta‐analysis aimed to evaluate the clinical efficacy of adjunctive biological agents in combination with OFD compared to OFD alone for the treatment of suprabony periodontal defects. The analysis of nine controlled clinical trials—eight being randomised—involving a total of 303 patients demonstrated statistically significant and clinically relevant improvements favouring the adjunctive use of biologics. Specifically, EMD and PRF both resulted in significantly lower residual PD (MD: EMD = −0.89 mm, PRF = −0.42 mm) and CAL (MD: EMD = −1.32 mm, PRF = −0.69 mm) compared to OFD alone. The only study employing HA also demonstrated similar improvements (PD = −0.74 mm, CAL = −1.23 mm). EMD was also associated with significantly lower residual REC (MD: −0.61 mm).

Clinical improvements in this review align with prior studies on regenerative therapy for intrabony defects (Bhutda and Deo 2013; Cortellini et al. 2022), but the inter‐group differences were smaller, likely because suprabony defects lack the bony walls needed for optimal regeneration (Polimeni et al. 2006; Wikesjö et al. 2008). Moreover, our findings are consistent with the systematic review by Graziani et al. (2014) evaluating EMD application in suprabony defects and reporting mean CAL gains of 1.2 and PD reductions of approximately 1.2 mm. Notably, our analysis incorporated the same three studies along with an additional trial by Iorio‐Siciliano et al. (2021) resulting in a pooled lower residual PD of 0.89 mm. The similarity of these results confirms that biologics can significantly enhance clinical outcomes. Further supporting our results, Graziani et al. (2012) and Jepsen et al. (2020), evaluating regenerative materials in intrabony and furcation defects, respectively, reported comparable soft‐tissue improvements, reinforcing the clinical value of biologics across various periodontal defect morphologies. Unlike Graziani et al. (2014), our study did not include the two subgroups from the Yilmaz et al. (2003)'s study, specifically those with baseline probing pocket depths of 1–3 mm treated with either intracrevicular incision or reverse bevel incision. These shallow periodontal sites were excluded because probing depths ≤ 3 mm generally represent clinically healthy or minimally affected sites, either in pristine or reduced periodontium, and therefore do not meet the criteria for true suprabony periodontal defects. This exclusion allowed us to focus specifically on clinically relevant suprabony defects.

Importantly, the present analysis demonstrated significantly less residual REC in groups treated with EMD compared to OFD alone. Limiting postoperative REC has crucial implications for aesthetics, patient comfort and overall satisfaction. This beneficial effect likely results from the biologically mediated wound healing induced by EMD, involving enhanced fibroblast proliferation, angiogenesis and soft‐tissue density (Miron et al. 2017; Wennström and Lindhe 2002).

PRF serves as a fibrin scaffold containing platelets, leukocytes and key growth factors that promote wound healing and tissue regeneration. In this analysis, PRF showed greater improvements in residual PD and CAL than OFD alone, but did not significantly reduce REC like EMD. These differences may be due to variations in their physical properties and regenerative mechanisms, highlighting the need for further comparative studies on PRF and EMD in periodontal healing (Miron et al. 2017).

Bone‐level changes across the included studies were modest and inconsistent. This validates the limited regenerative potential of suprabony defects due to their horizontal morphology, which inherently offers minimal structural support for bone regeneration compared to intrabony defects (Polimeni et al. 2006; Saleh, Mallala, et al. 2024).

Although pocket closure is identified by the AAP and EFP as the primary therapeutic goal (Sanz et al. 2020; Chapple et al. 2018), only one study (Di Tullio et al. 2013) specifically assessed pocket closure, while others focused on indirect parameters such as PD or CAL. This underscores the lack of standardisation in outcome reporting and the need for future studies to align with established clinical endpoints.

Several methodological limitations should be considered when interpreting these findings. First, substantial heterogeneity was observed among the included studies, particularly regarding residual CAL and REC. Variability in surgical approaches, defect characteristics, application methods of the biologics and follow‐up durations across trials likely contributed to this heterogeneity. Additionally, the overall risk of bias assessment indicated ‘some concerns’, mainly due to deviations from intended interventions in several trials. Sensitivity analysis revealed that certain studies, notably those of Yilmaz et al. (2003) and Joseph et al. (2014), substantially influenced outcomes, highlighting their impact on result stability. The quality of evidence assessed via the GRADE approach was low to very low, primarily due to inconsistency and risk of bias, underscoring the necessity for cautious interpretation and emphasising the need for standardised protocols and robust methodological designs in future trials.

Since most included studies reported only full‐mouth PI and BOP values, extraction of site‐specific data was not possible. Therefore, full‐mouth scores were included, acknowledging that they are less precise than site‐specific measures and that the results should be interpreted in light of this limitation.

Adjunctive biologics' effectiveness in suprabony periodontal defects may vary by tooth location, as anterior/posterior sites and maxillary/mandibular regions have different anatomical features, including bone density and root morphology, that can influence treatment outcomes. In this review, only Yilmaz et al. (2003) reported the treated arch, and only Jentsch and Purschwitz (2008) and Yilmaz et al. (2003) provided data on anterior and posterior regions. However, limited data prevented detailed analysis, although these anatomical differences remain important for predicting treatment success.

Another critical variable is the choice of the incision technique. Incisions and flap designs, such as intracrevicular incisions, PPT or reverse bevel incisions, can influence clinical outcomes by affecting wound stability, soft‐tissue preservation and blood supply. Minimally invasive designs that preserve the interdental papilla and maintain flap integrity may facilitate improved healing, especially in aesthetically sensitive areas (Cortellini et al. 1995).

This review shows that adjunctive biological agents can significantly improve PD and CAL in suprabony periodontal defects, suggesting that they could be routinely incorporated into clinical practice to enhance outcomes. However, since these improvements may reflect repair rather than true regeneration due to a lack of histological confirmation, the evidence remains limited. Further research is needed to clarify their long‐term effectiveness and regenerative potential and to establish standardised protocols for their use.

Future research should prioritise high‐quality, adequately powered RCTs with rigorous methodological standards, including standardised definitions of suprabony defects, surgical techniques such as PPT and consistent application protocols of biologics. Additionally, incorporating patient‐centred outcomes, such as tooth survival, economic considerations and long‐term patient satisfaction, should be considered (Saleh, Dias, and Kumar 2024). Since Graziani et al. (2014)'s review, published over a decade ago, few studies have been conducted using biologics in treating suprabony defects despite the growing use of biologics in periodontal therapy. This stresses the need for more well‐designed studies to strengthen the provided evidence.

Clinicians should interpret these findings pragmatically, noting that while biologics offer moderate improvements, they are best used as adjuncts within comprehensive periodontal treatment. Decisions should consider patient factors, defect morphology, aesthetic needs, cost effectiveness and preferences.

## 5 Conclusion

Within its limitations, this systematic review and meta‐analysis demonstrated that the adjunctive use of biological agents (EMD, PRF and HA) with OFD provides statistically significant and clinically relevant superior results in terms of residual PD, CAL and REC in the treatment of suprabony periodontal defects compared to OFD alone. However, because of the substantial heterogeneity among studies and methodological limitations, including inconsistent radiographic assessments, these findings should be interpreted cautiously. High‐quality RCTs with standardised protocols, consistent radiographic evaluations and longer‐term follow‐ups are essential to confirm these results and establish clearer clinical guidelines.

## Author Contributions

**S.B.:** conceptualisation, project administration, methodology, investigation, writing – original draft, writing – review and editing. **P.H.:** data curation, formal analysis, writing – original draft, writing – review and editing. **H.S.:** investigation, writing – review and editing. **AE.A.:** methodology, writing – review and editing. **L.P.‐B.:** writing – original draft, writing – review and editing. **S.A.E.:** writing – original draft, writing – review and editing. **H.L.W.:** supervision, methodology, writting – review and editing. **M.H.A.S.:** supervision, methodology, writing – review and editing.

## Funding

The authors have nothing to report.

## Conflicts of Interest

The authors declare no conflicts of interest.

## Supporting information


**Data S1:** jcpe70078‐sup‐0001‐PRISMA_2020_checklist.pdf.

**Data S2:** jcpe70078‐sup‐0002‐Supplementary_Material.docx.

## Data Availability

The data that support the findings of this study are available from the corresponding author upon reasonable request.

## Methods and Materials

### Meta‐Analysis

Five separate meta‐analyses were conducted for the three main outcomes, namely PD, CAL and REC. In the first one, studies were stratified by the type of biological agent, and no overall effect size was calculated, as pooling studies using different biologics would not yield clinically or conceptually meaningful results. In the second and third meta‐analyses, studies using EMD and PRF separately were stratified according to their follow‐up time. Finally, in the fourth and fifth meta‐analyses, studies using EMD and PRF were stratified based on their flap design in two separate analyses. All these analyses were conducted on post‐intervention (residual) values due to the availability of data. Standard deviations were adjusted for studies with a split‐mouth design by calculating the correlation coefficient between baseline and follow‐up values (Higgins et al. 2019). Both common‐effect (fixed‐effects) and random‐effects models were applied and presented in the forest plots; however, due to substantial heterogeneity among the studies and differences in their interventions, only the results from the random‐effects model were considered for inclusion in the final reported results (Dettori et al. 2022). In studies with multiple experimental arms, either irrelevant arms were excluded or eligible arms were combined into a single arm (Higgins et al. 2019). Also, when the study included independent pairs of treatment and control, they were treated as separate trials published within a single report. Because of inconsistencies in the reporting and definition of treatment sites across studies, all analyses were conducted at the patient‐level unit of allocation. For PD, CAL and REC, meta‐analysis was performed using the mean difference (MD) as the effect size measure; however, the standardised mean difference (SMD) was used for bleeding and plaque indices due to inconsistencies in the metrics used among the included studies. Finally, sensitivity analysis was performed using a leave‐one‐out approach.

## Results

### Baseline and Treatment Characteristics of the Suprabony Defects

All studies employed OFD alone as the control, although the surgical technique of getting access differed across studies. While most adopted traditional access flaps (Debnath and Chatterjee 2018; Jentsch and Purschwitz 2008; Joseph et al. 2014; Kizildağ et al. 2018; Yilmaz et al. 2003), others applied SPPT/MPPT (Di Tullio et al. 2013; Iorio‐Siciliano et al. 2021; Subramanian et al. 2025; Vela et al. 2024). Systemic antibiotics were used inconsistently, with some studies administering amoxicillin or amoxicillin–clavulanic acid postoperatively (Di Tullio et al. 2013; Joseph et al. 2014; Kizildağ et al. 2018; Yilmaz et al. 2003).

Studies using PRF as an adjunctive biologic employed different preparation methods. In the study by Kizildağ et al. (2018), the PRF clot was placed in a PRF box, where it was compressed into a membrane while preserving its hydration, and the serum exudate was collected separately. Subramanian et al. (2025) prepared PRF by separating the clot from the red corpuscle base using sterile tweezers and scissors. Similarly, Joseph et al. (2014) isolated the PRF gel from the red blood cell layer using sterile stainless‐steel scissors, and the PRF membrane was obtained by pressing the clot between two pieces of surgical gauze, which was then placed over the PRF gel. Debnath and Chatterjee (2018) added an activator containing 0.1% gluconate to the PRF gel and mixed it for 9–10 times to obtain the PRFM gel. All studies that used EMD employed Emdogain (Straumann), except for Yilmaz et al. (2003)'s, which used BIORA AB. Additionally, Vela et al. (2024) used cross‐linked HA gel (hyaDENT BG, Bioscience).

#### Gingival Recession

##### Enamel Matrix Derivative

All three studies evaluating EMD and reporting REC (Di Tullio et al. 2013; Iorio‐Siciliano et al. 2021; Yilmaz et al. 2003) reported postoperative increases in REC in both test and control groups. However, all these studies consistently found significantly smaller increases in the EMD groups compared to controls (*p* < 0.05), suggesting a protective effect of EMD against gingival recession. Additionally, Iorio‐Siciliano et al. (2021) and Di Tullio et al. (2013) demonstrated a significantly lower residual REC in EMD than in control. While residual REC varied from 1.00 to 4.36 mm, primarily influenced by the baseline REC, the REC increase was reported to be between 0.5 and 0.68 mm consistently. Jentsch and Purschwitz (2008) did not assess REC.

##### Platelet‐Rich Fibrin

Only two of the included studies evaluating PRF (Debnath and Chatterjee 2018; Joseph et al. 2014; Kizildağ et al. 2018; Subramanian et al. 2025) reported REC (Joseph et al. 2014; Subramanian et al. 2025). While both studies found no significant difference in residual REC between groups, Subramanian et al. (2025) also found no significant increase in REC in PRF‐treated sites (*p* = 0.138). On the contrary, sites treated with OFD demonstrated a significant REC increase (*p* = 0.004).

##### Hyaluronic Acid

At 12 months, REC changes in the test group were minimal and not significant (0.08 ± 0.76, *p* = 0.6749), while the control group showed a significant increase (0.74 ± 1.03, *p* < 0.001). Moreover, intergroup comparison confirmed a significantly lower residual REC in PRF plus OFD compared to OFD alone (1.08 ± 1.36 vs. 1.69 ± 1.25; *p* < 0.0001).

#### Bone‐Level Changes

Radiographic or clinical bone‐level (BL) changes were reported in four studies, although the findings were not always consistent. Yilmaz et al. (2003) reported a radiographic BL gain of 0.10 mm in the test group treated with EMD compared to a loss of 0.17 mm in the control group treated with intracrevicular incision (*p* = 0.093), and a radiographic BL gain of 0.07 mm in the test group treated with EMD compared to a loss of 0.07 mm in the control group treated with reverse bevel incision (*p* = 0.484), none of which demonstrated a significant difference. Di Tullio et al. (2013) also did not observe significant differences in BL changes between groups. Kizildağ et al. (2018) reported significantly higher levels of bone morphogenic protein 2 (BMP‐2) and insulin‐like growth factor 1 (IGF‐1) secretion in the test group treated with OFD plus PRF, which suggests enhanced bone regeneration potential, even though direct BL measurements were not mentioned. In the study done by Joseph et al. (2014), they measured the relative crestal height. No statistically significant difference was observed either between the baseline and the follow‐up in the control and test groups or between the groups.

#### Bleeding and Plaque Indices

BOP and plaque scores were reduced from baseline in all included studies, consistent with improved oral hygiene and postoperative care. However, differences between the test and control groups were generally minimal. Reductions in FMPS and FMBS were observed across the board, but only a minority of studies demonstrated statistically significant superiority in the test groups. This suggests that while regenerative interventions enhance clinical healing, their influence on plaque control and bleeding may be only secondary to effective debridement and maintenance.

Among the studies (Di Tullio et al. 2013; Iorio‐Siciliano et al. 2021; Vela et al. 2024), reductions in FMBS and FMPS were significant within groups but broadly equivalent between arms, underscoring that hygiene compliance and professional maintenance remain central. In studies with biochemical endpoints (Kizildağ et al. 2018), inflammatory scores were uniformly improved but not significantly different across interventions, reflecting the systemic benefits of OFD itself.

#### Pocket Closure

Out of nine included studies, only one reported pocket closure. Di Tullio et al. (2013) observed a significantly higher proportion of sites achieving pocket closure (PD ≤ 3 mm) in the test group compared with the control (85% vs. 69%, *p* < 0.01). When stratified by baseline depth, closure occurred in 94.5% versus 84.2% of moderate pockets (4–6 mm, *p* < 0.05) and 57.7% versus 40.6% of deep pockets (≥ 7 mm, *p* < 0.01), favouring the test group. No residual deep pockets (PD ≥ 7 mm) were reported in either group.

### Meta‐Analysis

#### Probing Depth

##### Enamel Matrix Derivative

Stratification by follow‐up duration revealed substantial heterogeneity in both the 8‐month (*I* ^2^ = 92.4%, *τ* ^2^ = 0.84, *p* = 0.0003) and 12‐month (*I* ^2^ = 88.9%, *τ* ^2^ = 0.26, *p* = 0.0001) subgroups (Figure 2B). A significantly lower residual PD in favour of EMD was observed at 12 months (−1.04, 95% CI: −1.65 to −0.42, *p* = 0.0009), whereas the 8‐month subgroup did not show a significant difference between the EMD + OFD and control groups (−0.67, 95% CI: −1.99 to 0.65, *p* = 0.3203). The difference in effect size between the two follow‐up periods was not statistically meaningful (*p* = 0.6227).

##### Platelet‐Rich Fibrin

In the PRF sub‐analysis, a significantly lower PD was found at 9 months (−0.54, 95% CI: −0.81 to −0.27, *p* < 0.0001), while the 6‐month subgroup did not reach statistical significance (−0.37, 95% CI: −1.01 to 0.26, *p* = 0.2466). The difference between the two timepoints was not statistically significant (*p* = 0.6379) (Figure 2D).

#### Clinical Attachment Level

##### Enamel Matrix Derivative

A separate sub‐analysis of studies using EMD stratified according to the follow‐up duration (Figure 3B) showed substantial heterogeneity in the 12‐month subgroup (*I* ^*2*^ = 89.6%, *τ* ^2^ = 0.71, *p* < 0.0001). Despite this, the results consistently favoured the biologic groups, with both follow‐up periods demonstrating significantly lower residual CAL values compared to OFD alone (8 months: –1.09, 95% CI: −2.09 to −0.09, *p* = 0.0331; 12 months: –1.41, 95% CI: −2.41 to −0.42, *p* = 0.0054). Moreover, the effect was not significantly different between the two follow‐ups (*p* = 0.6533).

##### Platelet‐Rich Fibrin

A sub‐analysis of studies using PRF stratified by follow‐up duration (Figure 3D) showed a significantly lower CAL in favour of the biologic group at 6 months (−0.73, 95% CI: −1.31 to −0.15, *p* = 0.0132), whereas the effect was not statistically significant at 9 months (−0.56, 95% CI: −1.13 to 0.01, *p* = 0.0542). However, the difference in treatment effect between the two follow‐up periods was not significant (*p* = 0.6802).

#### Recession Depth

##### Enamel Matrix Derivative

When stratified by follow‐up duration, EMD showed a significantly lower REC at 12 months (−0.65, 95% CI: −0.95 to −0.35, *p* < 0.0001), whereas no significant difference was found in the 8‐month subgroup (−0.37, 95% CI: −1.07 to 0.33, *p* = 0.3039). The difference in treatment effect between the two time points was not statistically significant (*p* = 0.4719). Subgroup analysis by the surgical technique (Figure 4) showed a significantly lower REC when EMD was used in conjunction with PPT (−0.65, 95% CI: −0.95 to −0.35, *p* < 0.001), while no significant benefit was seen with conventional OFD (−0.37, 95% CI: −1.07 to 0.33, *p* = 0.3039). The between‐technique comparison did not reveal a statistically meaningful difference (*p* = 0.4719).

##### Platelet‐Rich Fibrin

Only two studies contributed to the PRF subgroup analysis—one reporting outcomes at 6 months and the other at 9 months, with one applying conventional OFD and the other PPT. Neither study showed a statistically significant difference in post‐intervention REC between the biologic and control groups.

#### Sensitivity Analysis

As presented in Table 3, the results of CAL, PD and REC meta‐analyses remained consistent across all iterations in EMD, indicating that the findings were generally stable. However, the overall effect estimates of PRF on CAL and PD changed and became statistically insignificant when Kizildağ et al. (2018) and Joseph et al. (2014) were excluded from the analysis, respectively. Changes in the subgroup analysis results following the leave‐one‐out analysis are presented in the Appendix. The subgroup analysis of the effect of EMD on PD and CAL, stratified by surgical technique, was sensitive to the omission of individual studies. Specifically, excluding Yilmaz et al. (2003) rendered the effect on CAL non‐significant and the effect on PD significant in the EMD + conventional OFD subgroup. Similarly, omitting Kizildağ et al. (2018) rendered the effect on CAL in the conventional OFD + PRF subgroup non‐significant.

#### Plaque and Bleeding Indices

Full‐mouth bleeding score (FMBS), bleeding on probing (BOP) and bleeding index were pooled together to calculate the effect of adjunct application of biologics (Löe 1967). As presented in Figure S3, neither EMD nor PRF had a statistically significant effect on bleeding (EMD: Hedge's *g* = −0.33, 95% CI: −0.81 to 0.15, *p* = 0.1771; PRF: Hedge's *g* = −0.32, 95% CI: −0.97 to 0.33, *p* = 0.3358). Also, there was no significant difference between the different subgroups (*p* = 0.7588). Similarly, regarding plaque indices, full‐mouth plaque score (FMPS) and plaque index (references please) were pooled in a meta‐analysis, where no significant effect of either EMD (Hedge's *g* = 0.2, 95% CI: −0.45 to 0.48, *p* = 0.9447) or PRF (Hedge's *g* = −0.31, 95% CI: −0.76 to 0.14, *p* = 0.1727) was detected, as well as the difference between subgroups (*p* = 0.6103) (Figure S1).

#### Quality of Evidence Analysis (GRADE Analysis)

As presented in Table S3, the quality of evidence for CAL gain was rated as ‘very low’, while the evidence for REC increases and PD reduction was rated as ‘low’. The primary factors contributing to the downgrading of the evidence of quality were inconsistency due to substantial heterogeneity across studies and the risk of bias in the included trials.
