# Supplementary material for: Efficacy of Biologics for Surgical Treatment of Periodontal Suprabony Defects: A Systematic Review and Meta‐Analysis of Controlled Clinical Trials
Source: J Clin Periodontol. 2026 Jan 14;53(5):735–51. doi: 10.1111/jcpe.70078 (PMC13086543; doi:10.1111/jcpe.70078)
Supplement: Supplementary file 2 — Data S2: jcpe70078‐sup‐0002‐Supplementary_Material.docx. [file JCPE-53-735-s002.docx]

**Table S1.** Search queries.

| ***Data Base*** | ***Date*** | ***Search Query*** | ***Result*** |
| --- | --- | --- | --- |
| PubMed | June  2025 | ("Guided Tissue Regeneration"[MeSH Terms] OR "guided tissue regeneration, periodontal"[MeSH Terms] OR "Periodontal Regeneration"[All Fields] OR "Surgical Flaps"[MeSH Terms] OR "Access"[All Fields] OR "Widman"[All Fields] OR "Dental Papilla"[MeSH Terms] OR "Papilla Preservation"[All Fields] OR "GTR"[All Fields] OR "Guided Tissue Regeneration"[All Fields] OR "Periodontal Regeneration"[All Fields] OR "periodontal flap"[All Fields] OR "periodontal surgery"[All Fields]) AND ("Dental Enamel Proteins"[Title/Abstract] OR "Amelogenin"[Title/Abstract] OR "EMD"[Title/Abstract] OR "enamel matrix derivative"[Title/Abstract] OR "enamel matrix derivatives"[Title/Abstract] OR "Dental Enamel Proteins"[MeSH Terms] OR "enamel matrix protein*"[Title/Abstract] OR "autologous blood derived product*"[Title/Abstract] OR "platelet-rich plasma"[Title/Abstract] OR "platelet-rich fibrin"[Title/Abstract] OR "platelet-rich fibrin"[Title/Abstract] OR "platelet-rich plasma"[Title/Abstract] OR "leukocyte-platelet-rich fibrin"[Title/Abstract] OR "plasma rich in growth factors"[Title/Abstract] OR "PRP"[All Fields] OR "PRF"[Title/Abstract] OR "L-PRF"[Title/Abstract] OR "A-PRF"[Title/Abstract] OR "PRGF"[Title/Abstract] OR "platelet-derived growth factor"[Title/Abstract] OR "PDGF"[Title/Abstract] OR "recombinant human platelet-derived growth factor BB"[Title/Abstract] OR "rhBMP-2"[Title/Abstract] OR "FGF"[Title/Abstract] OR "fibroblast growth factor"[Title/Abstract] OR "Bone morphogenetic protein"[Title/Abstract] OR "BMP"[Title/Abstract] OR "growth and differentiation factor 5"[All Fields] OR "GDF-5"[Title/Abstract] OR "teriparatide"[Title/Abstract] OR "PTH1-34"[Title/Abstract] OR "Hyaluronic Acid"[Title/Abstract] OR "HA"[Title/Abstract] OR "Biologics"[Title/Abstract]) AND ("suprabony defect*"[All Fields] OR "supra bony defect*"[All Fields] OR "supra alveolar defect*"[All Fields] OR "supra alveolar defect*"[All Fields] OR "horizontal defect*"[All Fields] OR "horizontal bone loss"[All Fields] OR "horizontal periodontal defect*"[All Fields] OR "attachment loss"[All Fields] OR "periodontal bone loss"[All Fields]) | 362 |
| Scopus | June  2025 | ALL ( ( "Guided Tissue Regeneration" OR "Periodontal Regeneration" OR "Surgical Flaps" OR "Access" OR "Widman" OR "Dental Papilla" OR "Papilla Preservation" OR "GTR" OR "periodontal flap" OR "periodontal surgery" ) ) AND TITLE-ABS-KEY ( ( "Dental Enamel Protein*" OR "Amelogenin" OR "EMD" OR "enamel matrix derivative*" OR "autologous blood-derived product" OR "platelet-rich plasma" OR "platelet-rich fibrin" OR "leukocyte--platelet-rich fibrin" OR "plasma rich in growth factors" OR "PRP" OR "PRF" OR "L-PRF" OR "A-PRF" OR "PRGF" OR "platelet-derived growth factor" OR "PDGF" OR "recombinant human platelet-derived growth factor BB" OR "rhBMP-2" OR "FGF" OR "fibroblast growth factor" OR "Bone morphogenetic protein" OR "BMP" OR "growth and differentiation factor 5" OR "GDF-5" OR "teriparatide" OR "PTH1-34" OR "Hyaluronic Acid" OR "HA" OR "Biologics" ) ) AND ALL ( ( "suprabony defect*" OR "supra-bony defect*" OR "supra alveolar defect*" OR "supra-alveolar defect*" OR "horizontal defect*" OR "horizontal bone loss" OR "horizontal periodontal defect*" OR "supragingival defect*" OR "attachment loss" OR "periodontal bone loss" ) ) | 611 |
| Embase | June  2025 | ('guided tissue regeneration'/exp OR 'guided tissue regeneration, periodontal'/exp OR 'surgical flap'/exp OR access:ti,ab OR widman:ti,ab OR 'dental papilla'/exp OR 'papilla preservation':ti,ab OR gtr:ti,ab OR 'guided tissue regeneration':ti,ab OR 'periodontal regeneration':ti,ab OR 'periodontal flap':ti,ab OR 'periodontal surgery':ti,ab) AND ('dental enamel protein' OR amelogenin:ti,ab OR emd:ti,ab OR 'enamel matrix derivative':ti,ab OR 'enamel matrix derivatives':ti,ab OR 'enamel matrix protein*':ti,ab OR 'autologous blood derived product*':ti,ab OR 'platelet rich plasma':ti,ab OR 'platelet rich fibrin':ti,ab OR 'leukocyte platelet rich fibrin':ti,ab OR 'plasma rich in growth factors':ti,ab OR prp:ti,ab OR prf:ti,ab OR 'l prf':ti,ab OR 'a prf':ti,ab OR prgf:ti,ab OR 'platelet derived growth factor':ti,ab OR pdgf:ti,ab OR 'recombinant human platelet derived growth factor bb':ti,ab OR 'rhbmp 2':ti,ab OR fgf:ti,ab OR 'fibroblast growth factor':ti,ab OR 'bone morphogenetic protein':ti,ab OR bmp:ti,ab OR 'growth and differentiation factor 5':ti,ab OR 'gdf 5':ti,ab OR teriparatide:ti,ab OR 'pth1 34':ti,ab OR 'hyaluronic acid':ti,ab OR ha:ti,ab OR biologics:ti,ab) AND ('suprabony defect*':ti,ab OR 'supra-bony defect*':ti,ab OR 'supra alveolar defect*':ti,ab OR 'supra-alveolar defect*':ti,ab OR 'horizontal defect*':ti,ab OR 'horizontal bone loss':ti,ab OR 'horizontal periodontal defect*':ti,ab OR 'supragingival defect*':ti,ab OR 'attachment loss':ti,ab OR 'periodontal bone loss':ti,ab) | 81 |
| Web of Science | June  2025 | TS=("guided tissue regeneration" OR "guided tissue regeneration, periodontal" OR "periodontal regeneration" OR "surgical flaps" OR "access" OR "widman" OR "dental papilla" OR "papilla preservation" OR "GTR" OR "periodontal flap" OR "periodontal surgery")  AND  TS=("dental enamel proteins" OR "amelogenin" OR "EMD" OR "enamel matrix derivative" OR "enamel matrix derivatives" OR "enamel matrix protein*" OR "autologous blood-derived product*" OR "platelet-rich plasma" OR "platelet-rich fibrin" OR "platelet rich fibrin" OR "platelet rich plasma" OR "leukocyte platelet-rich fibrin" OR "plasma rich in growth factors" OR "PRP" OR "PRF" OR "L-PRF" OR "A-PRF" OR "PRGF" OR "platelet-derived growth factor" OR "PDGF" OR "recombinant human platelet-derived growth factor BB" OR "rhBMP-2" OR "FGF" OR "fibroblast growth factor" OR "bone morphogenetic protein" OR "BMP" OR "growth and differentiation factor 5" OR "GDF-5" OR "teriparatide" OR "PTH1-34" OR "hyaluronic acid" OR "HA" OR "biologics")  AND  TS=("suprabony defect*" OR "supra-bony defect*" OR "supra alveolar defect*" OR "supra-alveolar defect*" OR "horizontal defect*" OR "horizontal bone loss" OR "horizontal periodontal defect*" OR "supragingival defect*" OR "attachment loss" OR "periodontal bone loss") | 75 |

**Table S2**. Excluded studies

| *Excluded studies* | *Reasons* |
| --- | --- |
| Moreno Rodríguez et al. (1) | Case series |
| Gamal et al. (2) | Infrabony defects |

**Table S3.** Quality of evidence according to the GRADE framework.

| *Certainty assessment* | | | | | | | *№ of patients* | | *Effect* | | *Certainty* | *Importance* |
| --- | --- | --- | --- | --- | --- | --- | --- | --- | --- | --- | --- | --- |
| *№ of studies* | ***Study design*** | ***Risk of bias*** | ***Inconsistency*** | ***Indirectness*** | ***Imprecision*** | ***Other considerations*** | ***OFD + Biologic*** | ***OFD*** | ***Relative (95% CI)*** | ***Absolute (95% CI)*** |  |  |
| PD (EMD) (follow-up: range 8 months to 12 months; assessed with: mm) | | | | | | | | | | | | |
| 4 | randomised trials | not serious | very serious | not serious | serious | none | 106 | 88 | - | MD 0.89 mm lower (1.45 lower to 0.32 lower) | ⨁◯◯◯ Very low | IMPORTANT |
| PD (PRF) (follow-up: range 6 months to 9 months; assessed with: mm) | | | | | | | | | | | | |
| 4 | randomised and non-randomised trials | serious | not serious | not serious | serious | none | 69 | 54 | - | MD 0.42 mm lower (0.72 lower to 0.12 lower) | ⨁⨁◯◯ low | IMPORTANT |
| CAL (EMD) (follow-up: range 8 months to 12 months; assessed with: mm) | | | | | | | | | | | | |
| 4 | randomised trials | not serious | very serious | not serious | not serious | none | 106 | 88 | - | MD 1.32 mm lower (2.00 lower to 0.64 lower) | ⨁⨁◯◯ low | CRITICAL |
| CAL (PRF) (follow-up: range 6 months to 9 months; assessed with: mm) | | | | | | | | | | | | |
| 4 | randomised and non-randomised trials | serious | not serious | not serious | serious | none | 69 | 54 | - | MD 0.69 mm lower (1.05 lower to 0.33 lower) | ⨁⨁◯◯ low | CRITICAL |
| REC (EMD) (follow-up: range 8 months to 12 months; assessed with: mm) | | | | | | | | | | | | |
| 3 | randomised trials | not serious | not serious | not serious | serious | none | 81 | 74 | - | MD 0.61 mm lower (0.88 lower to 0.33 lower) | ⨁⨁⨁◯ moderate | IMPORTANT |
| REC (PRF) (follow-up: range 6 months to 9 months; assessed with: mm) | | | | | | | | | | | | |
| 2 | randomised and non-randomised | not serious | not serious | serious | very serious | none | 45 | 30 | - | MD 0.07 mm lower (0.42 lower to 0.29 higher) | ⨁◯◯◯ very low | CRITICAL |
| Bleeding indices (EMD) (follow-up: range 8 months to 12 months; assessed with: mm) | | | | | | | | | | | | |
| 3 | randomised trials | not serious | not serious | serious | very serious | none | 81 | 63 | - | SMD 0.33 SD lower (0.81 lower to 0.15 higher) | ⨁◯◯◯ very low | IMPORTANT |
| Bleeding indices (PRF) (follow-up: range 6 months; assessed with: mm) | | | | | | | | | | | | |
| 2 | randomised trials | not serious | not serious | serious | very serious | none | 31 | 31 | - | SMD 0.32 SD lower (0.97 lower to 0.33 higher) | ⨁◯◯◯ very low | IMPORTANT |
| Plaque indices (EMD) (follow-up: range 8 months to 12 months; assessed with: mm) | | | | | | | | | | | | |
| 2 | randomised trials | not serious | not serious | serious | very serious | none | 56 | 49 | - | SMD 0.02 SD lower (0.45 lower to 0.48 higher) | ⨁◯◯◯ very low | IMPORTANT |
| Plaque indices (PRF) (follow-up: range 8 months to 12 months; assessed with: mm) | | | | | | | | | | | | |
| 3 | randomised trials | not serious | not serious | serious | very serious | none | 39 | 39 | - | SMD 0.31 SD lower (0.76 lower to 0.14 higher) | ⨁◯◯◯ very low | IMPORTANT |

**Figure S1.**

**Figure S2.** Risk of bias assessment of the non-randomized controlled clinical trials.

**Figure S3.** Risk of bias assessment of the Randomized controlled trials.

1.	Moreno Rodríguez JA, Ortiz Ruiz AJ, Caffesse RG. Supra-alveolar attachment gain in the treatment of combined intra-suprabony periodontal defects by non-incised papillae surgical approach. J Clin Periodontol. 2019;46(9):927–36.

2.	Gamal AY, Abdel-Ghaffar KA, Iacono VJ. Gingival crevicular fluid vascular endothelial cell growth factor and platelet-derived growth factor-BB release profile following the use of perforated barrier membranes during treatment of intrabony defects: a randomized clinical trial. J Periodontal Res. 2016;51(3):407–16.
